# Risk Factors for Developmental Dysplasia of the Hip: A Critical Analysis About an Unclear Relationship

**DOI:** 10.3390/jcm13226898

**Published:** 2024-11-16

**Authors:** Tamir Dib, Matteo Nanni, Ilaria Sanzarello, Giada Salvatori, Daniela Alessia Marletta, Biagio Zampogna, Danilo Leonetti

**Affiliations:** 1 BIOMORF Department of Biomedical, Dental, Morphological and Functional Images, University of Messina, A.O.U Policlinico “G. Martino”, Via Consolare Valeria 1, 98124 Messina, Italy; 2 IRCCS Sacro Cuore Don Calabria Negrar, Viale Luigi Rizzardi 4, 37024 Verona, Italy; 3 Operative Research Unit of Orthopaedic and Trauma Surgery, Fondazione Policlinico Universitario Campus Bio-Medico, Via Alvaro del Portillo 200, 00128 Rome, Italy; 4 Research Unit, Orthopaedic and Trauma Surgery, Università Campus Bio-Medico Di Roma, Via Alvaro del Portillo 21, 00128 Rome, Italy

**Keywords:** dysplasia, DDH, developmental dysplasia of the hip, risk factors, Graf, severity, relationship, Graf grade, screening

## Abstract

**Objective:** To evaluate the relationship between prenatal risk factors and developmental dysplasia of the hip using the Graf grade, and to identify the determinants of a higher Graf grade. **Materials and Methods:** A retrospective analysis of data from 112 newborns with DDH was conducted. The participants were selected on the basis of a DDH diagnosis using sonography. A total of 181 hips of patients with DDH were considered in our study group (Graf types IIa to IV), and the normal hips of those affected unilaterally were excluded from the analyses (43 participants were affected unilaterally). The risk factors considered included female sex, breech presentation, firstborn status, familiarity, association with other orthopedic abnormalities, and uterine packing, which includes factors such as twin pregnancy, macrosomia, and oligohydramnios. Binary logistic regression was used to analyze the relationship between these variables and the Graf type of DDH at presentation, which was defined using two groups: Graf types IIc–IV, which include unstable or decentered hips, and Graf types IIa and IIb, which encompass stable and centered hips. **Results:** The analyses revealed a significant protective role of the presence of other lower limb congenital malformations such as clubfoot, which was more closely associated with a stable form of DDH (OR = 0.26, *p* = 0.017), a significant association between the presence of mechanical risk factors in females with an unstable form of DDH (OR = 5.00, *p* = 0.042), a borderline significant protective role of breech presentation in females, which was more closely associated with a stable form of DDH (OR = 0.25, *p* = 0.054), and a borderline significant association between the presence of mechanical risk factors and an unstable form of DDH (OR = 4.28, *p* = 0.054). **Conclusions:** Prenatal risk factors may have a complex effect on the Graf grade in DDH. The protective effects of some factors in contrast with the increased risk associated with other factors suggest a possible relationship, with some prenatal risk factors affecting the severity of DDH. These findings may have implications for the early identification and management of DDH.

## 1. Introduction

Throughout the years, many factors have been considered to associate with DDH development, but whether these factors are coexistent or causative of DDH is not clear. Factors such as female sex, family history of DDH, and association with other orthopedic abnormalities of the lower limb could act in a non-mechanical way to influence the physiological evolution of the hip joint [1,2,3,4,5,6,7]. Other factors could mechanically affect the evolution of the hip joint, such as twin pregnancy, oligohydramnios, and macrosomia, referred to in this work as “uterine packing”; firstborn children could be affected by a similar mechanism. Breech presentation is another well-known factor associated with DDH that acts mechanically on the evolution of the hip joint [1,3,4,5,7,8,9,10,11,12]. The relationship between the presence of these prenatal risk factors and the severity of DDH remains underexplored to this day, and to our knowledge, only a few recent studies attempted to establish such a correlation [3,5]. Ultrasonographic diagnosis and grading according to the Graf method is currently the most used and accepted method to diagnose and grade DDH; this method is used to guide the choice of the method of intervention as well as to predict the outcome of treatment [2,10,11,12,13,14,15,16,17,18]. In many countries, the choice of the initial treatment type is based on the Graf grade as well as the decision to stop the treatment when the maturation of the hip joint is achieved and to escalate to a different, more aggressive treatment approach such as spica casting or surgical reduction when maturation is not achieved [1,2,3,4]. Our study aimed to evaluate the possible associations between these risk factors as predictive variables, with the severity of DDH defined according to the Graf type. We attempted to retrospectively analyze these factors for the DDH cases that were presented in our clinics between 2021 and 2024 to try to recognize the possible protectors or determinants of a higher Graf grade and, in turn, to reflect on the multifactorial nature of the pathogenesis of DDH. The data in our internal database were stored in a well-documented manner, which facilitated the creation of an adequate dataset for the current study and made a retrospective study design appropriate.

## 2. Materials and Methods

### 2.1. Data Collection

The data presented in this study were collected from the pediatric orthopedics unit of the UOC Ortopedia e Traumatologia in the G. Martino Policlinico Hospital of Messina, Italy, as well from pediatric orthopedics clinics in the regions of Veneto and Emilia-Romagna, Italy, between 2021 and 2024. A detailed anamnestic interview with the parents of the newborns was conducted by the doctors and residents of the department and included information about the course of the pregnancy and the presence of any breech positions during the third trimester, as well as oligohydramnios, macrosomia (birthweight and obstetric diagnosis), twin pregnancy, the firstborn status of the infant, and the mode of delivery. The parents were asked about a family history of DDH affecting first- and second-degree relatives. A thorough physical examination of the newborn was conducted by the attending pediatric orthopedist to detect and confirm the presence of any other congenital malformations of the lower extremity, such as metatarsus adductus and clubfoot. The parents presented the results of the ultrasonographic screening according to the Graf method, and these images were reassessed by the same attending doctors of the pediatric orthopedic unit to confirm the grading and diagnosis of DDH. 

### 2.2. Selection of Participants 

**Figure 1:**
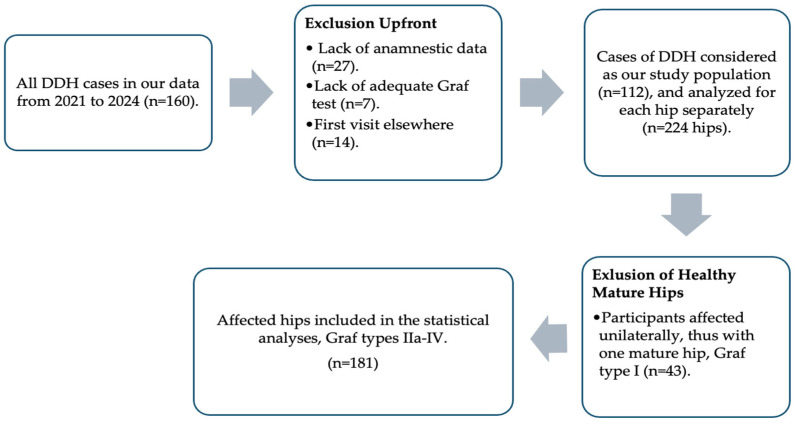
The selection process.

A total of 160 participants, all of which were diagnosed with DDH, were initially recognized in our retrospective collection of data from the summaries of the abovementioned visits; 48 participants were excluded upfront due to a lack of anamnestic data, inadequate ultrasonography according to the Graf method, mostly in multi-syndromic complex cases, or initiation of treatment or first assessment in a different institute. The data from the 112 participants that were eventually included in our study were considered separately for each hip, as both hips were not affected to the same extent in all the cases. Out of the 224 hips of the 112 participants included in the study, 181 hips were affected by DDH (i.e., Graf IIa or worse). The 43 hips that were Graf type I were the healthy hips of the participants that were unilaterally affected by DDH and were thus excluded from further analyses. All the participants in this study had no previous diagnosis of or treatment for DDH, and thus, the Graf type of the hips at presentation was always used to assess the influence of these prenatal risk factors on the very first Graf grading, which was the one used to determine the choice of initial treatment. See Figure 1 for the summary of the selection process. 

### 2.3. Data Curation 

Breech presentation, firstborn child, oligohydramnios, fetal macrosomia, and twin pregnancy were considered “mechanical risk factors”, and the last three were grouped under the term “uterine packing”, as they are thought to act through a similar mechanism. Familiarity and association with other orthopedic abnormalities were considered “non-mechanical risk factors”. The sex of the newborn was considered for the association with severity as well. The data about the grade, sex, and presence of risk factors were collected for each affected hip, and then the hips were divided into two groups based on the Graf grade: one group consisted of stable and centered hips (Graf IIa and IIb), and the other encompassed unstable or decentered hips (Graf IIc or worse). 

### 2.4. Statistical Analysis 

A binary logistic regression model was applied to assess the relationship between the severity of DDH at presentation based on the Graf grade in the two groups and the various prenatal factors considered regarding our data; three separate analyses were performed. The first (I) included all mentioned prenatal factors as well as the sex of the participant separately; this was carried out with the intention of recognizing the possible influence of any single factor on the Graf type. The second (II) considered associations between some factors and the sex of the participant; we chose breech presentation, as it was the most common risk factor in our study population, and the type of risk factor (mechanical or not) to try to recognize a possible influence of the mechanism of action of the risk factor on the Graf type. The third (III) considered the type of risk factor (mechanical or non-mechanical) and the presence of a risk factor of any type as a variable that could affect the Graf type in our participants; this was an attempt to recognize any possible influence of the presence of any risk factor or a certain type of risk factor (thus its mechanism of action) on the Graf grade. The statistical analyses were conducted using Python’s statsmodels package (version 0.14.1). 

## 3. Results

**Figure 2:**
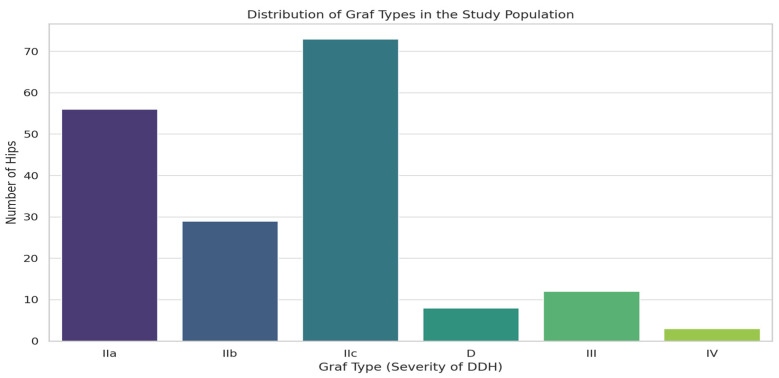
The distribution of Graf types in the study population.

Out of the 112 participants in the study, 88 were female, 24 were male, 69 were affected bilaterally at different grades of severity, 43 were affected unilaterally, 28 had left-sided DDH, and 15 had right-sided DDH. Thus, out of the 224 hips initially examined, only 181 hips were considered affected by DDH and were taken into consideration for our statistical analyses. A total of 33 (29%) participants had a breech presentation, and 10 of those were affected unilaterally. Thus, 56 hips were affected by DDH, 8 (7%) participants were firstborn, and 2 were affected unilaterally, and thus, 14 hips were affected. A total of eight (7%) participants were exposed to uterine packing, and six of those were affected unilaterally. Thus, 10 hips were affected, and 11 (10%) participants had a family history of DDH, 8 of which were unilaterally affected, and thus, 14 hips were affected, and 12 (10%) participants had other orthopedic abnormalities of the lower limb, 5 of which were unilaterally affected by DDH, and thus, 19 hips were affected. Out of the 88 females in the study, 37 were unilaterally affected; thus, 139 hips belonged to females affected by DDH. Moreover, 104 hips out of the original 224 had no risk factors at all when disregarding the sex of the newborn; 20 of these were male hips, 19 of them were affected by DDH (Graf IIa or more), and 11 (55%) were of grade IIc or worse. These 11 male hips had no risk factors at all. Considering the 84 hips that were not exposed to any risk factor other than sex and affected by DDH, 64 hips (76%) required treatment initially (grade IIb or more), and among these, there were 11 male hips (17%), all of which were grade IIc or worse, that were not exposed to a risk factor of any kind. A summary of the distribution of risk factors is presented in Table 1. The distribution of hips across different Graf types is shown in Figure 2 and is as follows: Graf Type IIa, 56 hips (31%); Graf Type IIb, 29 hips (16%); Graf Type IIc, 73 hips (40%); Graf Type D, 8 hips (4%); Graf Type III, 12 hips (7%); and Graf Type IV, 3 hips (2%). Thus, 85 hips were considered stable and centered, and 96 hips were considered unstable or decentered. 

The results of the binary logistic regression were as follows: The first analysis (I) showed no significant results for the sex of the participant, the presentation, familiarity, firstborn status, or the presence of any uterine packing. It did show a significant association between the presence of other orthopedic abnormalities of the lower limb and the centered and stable type of hips (OR = 0.26, *p* = 0.017), which suggests protection from a more severe Graf type. The second analysis (II), which included the interaction of the sex of the participant with other risk factors, showed nonsignificant results for males that had any mechanical risk factor or males that had a breech presentation, but showed a borderline significant association between females that had a breech presentation and a centered and stable type of hips (OR = 0.21, *p* = 0.054), indicating protection from a more severe Graf type, and a significant association between females that had any mechanical risk factor and the decentered or unstable type of hips (OR = 5.00, *p* = 0.042). The third analysis (III) did not show significant results as far as non-mechanical factors or any risk factor in general was considered, but a borderline significant association of mechanical risk factors and the decentered or unstable type of hips regardless of the sex of the participant was reported (OR = 4.28, *p* = 0.054). The complete results of all three binary logistic regressions are reported in Table 2.

## 4. Discussion

In this retrospective study, we documented 112 cases of DDH; 69 were bilateral (62%), 43 of the cases were unilateral, 28 were left-sided (25%), and 15 were right-sided (13%). This diverges somewhat from the literature and studies that report a higher rate of unilateral DDH, which is mostly left-sided [1,10,18,19]. We suspect that to be because the most common risk factor in our study population was breech presentation, which is a well-reported risk factor that would lead to malalignment of both hip joints, unlike the cases of cephalic presentation where the compression of the left hip against the maternal pelvis is the probable cause of malalignment [1]. Despite only 23 (a third) cases of a breech presentation out of the 69 bilateral cases in our study population, we note that 70% of those with a breech presentation had bilateral DDH, and the majority of those with unilateral DDH and a breech presentation (6\10) had a right-sided case. These findings suggest a true effect of the fetal presentation on the laterality of DDH, but it was not the sole cause of higher rates of bilateral DDH in our study population, which suggests that other possible factors affect the laterality of DDH. Although there was no significant association between the presence or absence of risk factors and a higher grade of Graf in the participants without any risk factor (20 male hips), we note that among the 20 male hips not exposed to any prenatal risk factor, 55% had a high Graf grade necessitating treatment. In total, 64 hips were not exposed to any risk factor (other than the sex of the newborn) and required treatment initially (Graf IIb or worse); these represent 76% of the hips affected by DDH and were not exposed to risk factors. We believe that although risk factors in general influence the development of DDH and that some of these factors could have an influence over the Graf grade in each subject. The fact that a high percentage of participants in our study were not exposed to prenatal risk factors but still required treatment for DDH highlights the fact that this congenital malformation is multifactorial and has a complex pathogenesis. A single causative factor, or even multiple causative factors or triggers, was not identified in many cases, and we noted that among these cases, a high percentage required treatment, and thus, a wide screening program could benefit these cases. The literature does mention that in many DDH cases, a risk factor is not identified, refs [1,10], yet in many countries, a screening program is based on the presence of risk factors or clinical signs that are also often absent, particularly in DDH hips with lower Graf grades [20,21,22,23]. The results of the statistical analyses demonstrated that mechanical risk factors, which included factors that would mechanically contribute to malalignment of the hip joint and, in turn, impair its evolution, are strongly associated with a higher Graf type. These factors included breech presentation, firstborn children, and uterine packing, which, in turn, included oligohydramnios, macrosomia, and twin pregnancy. These factors are well reported in the literature to be associated with an increased incidence of DDH [1,7,8,11]. Since the hypothesized mechanism of action of these factors is thought to be similar, we grouped them under the same “mechanical risk factors” category. The increased rates of higher Graf grades were found to be of borderline significance for mechanical risk factors when considered for both sexes (OR = 4.28, *p* = 0.054) and significant when considering females only (OR = 5.00, *p* = 0.042). The most common mechanical factor in our study population, breech presentation in females, was found to be associated with lower rates of higher Graf grades, and although this was only of borderline significance (OR = 0.21, *p* = 0.054), it does suggest that breech presentation may have a different mechanism of action relative to other factors that are considered to act mechanically on the alignment of the hip joint. In addition, in our clinical experience, we noted a relatively fast recovery and a good response to treatment in newborns with DDH that had a breech presentation. For females in particular, this mechanism of increased rates of DDH as well as the quick reduction and maturation of the hip joint could be explained by the increased joint laxity noted in females [6], which could underly the association with lower Graf grades in our results for this group. Of note, although the modality of delivery (vaginal or cesarian) could influence the effects of the presentation as a risk factor [5,7,8], we did not include this as a variable in our analysis, which should be considered when interpreting this result, as it might have had an effect on the borderline significance of these results and could mean that the real effect of breech presentation in females needs to be further explored. Moreover, although our data did not demonstrate this directly for each separate factor, we note that there might be some factors that, when considered in combination and in specific subgroups of the population, could in fact act as determinants of a higher Graf grade. This association is probably a complex one but could highlight the importance of screening for DDH, especially in some subgroups of the population, as well as the importance of anamnesis both for screening and for treatment initiation. Another important result of our analyses showed an association between the presence of other congenital orthopedic abnormalities of the lower limb and a lower Graf grade (OR = 0.26, *p* = 0.017). In our study population, these abnormalities were, for the most part, foot deformities, which included clubfoot and metatarsus adductus as the most common deformities that were reported. An association between DDH and these abnormalities is well reported in the literature, but the exact underlying mechanism is not clear [1,10,11,18]. The fact that another well-reported factor that is associated with DDH is correlated to a lower Graf grade further highlights the complex pathogenesis and the multifactorial etiology of this congenital malformation. 

### 4.1. Limitations 

In our study, a total of 112 participants were included, although we believe that including only cases of DDH and considering each single hip separately while excluding the healthy mature hips from our study population is an advantage of our study, the limited number of participants is a strong limitation in our case. We believe that more similarly structured studies with larger numbers of participants could indeed yield better and more significant results, particularly when considering subgroups of the populations, such as males or twins, and uterine packing in general, which were present in relatively small numbers in our study. Another limitation of our study to be taken into consideration is the lack of inclusion of the modality of delivery as a predictive variable for DDH severity. It was noted in some studies [5,7,8] that it could affect the rates of DDH presentation, and further studies might benefit from a consideration of this variable in their analysis. An important limitation regarding the presence of other orthopedic abnormalities and the apparent protection from a more severe Graf type is the fact that although most of the cases (11/12) affected the foot, these were not the same deformity, and thus, any conclusions about this relationship should be carefully drawn and probably further studied in a more specific manner.

### 4.2. Possible Clinical Implications 

Considering our results as well as the limitations of our study, we would like to carefully highlight the lack of highly significant results regarding the identification of determinants of a more severe Graf grade, which, in our opinion, underscores the importance of a general screening program for developmental dysplasia of the hip. The fact that some factors seemingly provide protection from severe DDH while others are seemingly correlated with a more severe type does not exclude the need for treatment and possibly necessitates early treatment in any case of DDH. Patients with more severe types could indeed benefit from earlier recognition since there could be a higher rate of treatment failure [1,2,3,4,5]; hips with the less severe DDH can benefit from an early diagnosis and treatment initiation as well. This is especially true when we note from our results that an important part of our study population that was not exposed to any risk factor had pathological hips that required treatment. In summary, we emphasize the importance of screening for DDH in newborns with prenatal risk factors on anamnesis, but the need for screening when “a protective” factor is noted or in the absence of risk factors should not be excluded. This multifaceted approach to the diagnosis and management of an orthopedic condition is well reported in the literature [24], often requiring multiple steps and modalities of evaluation, such as, in this case, anamnestic, clinical, and instrumental.

## 5. Conclusions

The results of this study could highlight the complex role of prenatal risk factors and their effect on the morphological maturity of the hip joint and its evolution, which could further support a multifactorial etiological model that underlies DDH development and the complexity of the interactions between these factors. Some of these factors showed protection from highly immature or unstable hips, while others were associated with less mature and unstable hips. These results suggest that these prenatal factors could correlate with Graf type and thus the severity of the malformation when considered in combination or in specific subgroups of the population. We cannot state clearly that some factors are clear protectors or determinants of a more severe form of DDH. These findings may have implications for the modality of screening, early diagnosis, treatment strategies, and overall management of developmental dysplasia of the hip, highlighting the importance of screening for DDH and the use of anamnestic, clinical, and instrumental tools in this context.

## Figures and Tables

**Table 1:** Risk factors and hips affected/exposed.

Risk Factor	Hips Exposed (%) *	Hips Affected (%) **
Breech presentation	66 (29%)	56 (31%)
First-born child	16 (7%)	14 (8%)
Uterine packing	16 (7%)	10 (5%)
Familiarity	22 (10%)	14 (8%)
Other orthopedic abnormalities	24 (10%)	19 (10%)
Female sex	176 (79%)	139 (77%)
No risk factors	104 (46%)	84 (46%)
Males without risk factors	20 (41%) ^1^	19 (45%) ^3^
Females without other risk factors	84 (48%) ^2^	65 (48%) ^4^

* % of the total study population. ** % of the total hips affected. ^1^ % of total males. ^2^ % of total females. ^3^ % of the total affected males. ^4^ % of the total affected females.

**Table 2:** The results of the statistical analysis.

	Predictor	*p*-Value	Odds Ratio	95% CL
I	Sex (male vs. female)	0.17	0.59	(0.33, 1.06)
	Presentation (breech vs. cephalic)	0.93	0.97	(0.43, 2.18)
	Familiarity	0.68	0.79	(0.25, 2.56)
	Other orthopedic abnormalities	0.017	0.26	(0.14, 0.50)
	Uterine packing	0.34	0.57	(0.21, 1.58)
	First-born status	0.56	1.27	(0.56, 2.86)
II	Female, breech	0.054	0.21	(0.07, 0.60)
	Male, breech	0.69	0.54	(0.02, 13.63)
	Female, mechanical	0.042	5.00	(1.85, 13.54)
	Male, mechanical	0.47	3.10	(0.34, 11.53)
III	Mechanical risk factors	0.054	4.28	(1.16, 11.29)
	Non-mechanical risk factors	0.58	1.57	(0.31, 7.92)
	Presence of any risk factor	0.11	0.30	(0.10, 0.87)

## Data Availability

The raw data supporting the conclusions of this article will be made available by the authors upon request.
